# Sepsis at ICU admission does not decrease 30-day survival in very old patients: a post-hoc analysis of the VIP1 multinational cohort study

**DOI:** 10.1186/s13613-020-00672-w

**Published:** 2020-05-13

**Authors:** Mercedes Ibarz, Ariane Boumendil, Lenneke E. M. Haas, Marian Irazabal, Hans Flaatten, Dylan W. de Lange, Alessandro Morandi, Finn H. Andersen, Guido Bertolini, Maurizio Cecconi, Steffen Christensen, Loredana Faraldi, Jesper Fjølner, Christian Jung, Brian Marsh, Rui Moreno, Sandra Oeyen, Christina Agwald Öhman, Bernardo Bollen Pinto, Ivo W. Soliman, Wojciech Szczeklik, Andreas Valentin, Ximena Watson, Tilemachos Zaferidis, Bertrand Guidet, Antonio Artigas, the VIP1 study, René Schmutz, Franz Wimmer, Philipp Eller, Michael Joannidis, Pieter De Buysscher, Nikolaas De Neve, Sandra Oeyen, Walter Swinnen, Bernardo Bollen Pinto, Paul Abraham, Leila Hergafi, Joerg C. Schefold, Ewelina Biskup, Petr Piza, Ioannis Taliadoros, Jesper Fjølner, Nilanjan Dey, Christoffer Sølling, Bodil Steen Rasmussen, Steffen Christensen, Xavier Forceville, Guillaume Besch, Herve Mentec, Philippe Michel, Philippe Mateu, Philippe Michel, Lucie Vettoretti, Jeremy Bourenne, Nathalie Marin, Max Guillot, Nadia Aissaoui, Cyril Goulenok, Nathalie Thieulot-Rolin, Jonathan Messika, Lionel Lamhaut, Bertrand Guidet, Cyril Charron, Alexander Lauten, Anna Lena Sacher, Thorsten Brenner, Marcus Franz, Frank Bloos, Henning Ebelt, Stefan J. Schaller, Kristina Fuest, Christian Rabe, Thorben Dieck, Stephan Steiner, Tobias Graf, Amir M. Nia, Christian Jung, Rolf Alexander Janosi, Patrick Meybohm, Philipp Simon, Stefan Utzolino, Tim Rahmel, Eberhard Barth, Christian Jung, Michael Schuster, Zoi Aidoni, Stavros Aloizos, Polychronis Tasioudis, Kleri Lampiri, Vasiliki Zisopoulou, Ifigenia Ravani, Evmorfia Pagaki, Angela Antoniou, Theodoros A. Katsoulas, Aikaterini Kounougeri, George Marinakis, Fotios Tsimpoukas, Anastasia Spyropoulou, Paris Zygoulis, Aikaterini Kyparissi, Manish Gupta, Mohan Gurjar, Ismail M. Maji, Ivan Hayes, Brian Marsh, Yvelynne Kelly, Andrew Westbrook, Gerry Fitzpatrick, Darshana Maheshwari, Catherine Motherway, Giovanni Negri, Savino Spadaro, Guisepepe Nattino, Matteo Pedeferri, Annalisa Boscolo, Simona Rossi, Giuseppe Calicchio, Lucia Cubattoli, Gabriella Di Lascio, Maria Barbagallo, Francesco Berruto, Daniela Codazzi, Andrea Bottazzi, Paolo Fumagalli, Giancarlo Negro, Giuseppe Lupi, Flavia Savelli, Giuseppe A. Vulcano, Roberto Fumagalli, Andrea Marudi, Ugo Lefons, Rita Lembo, Maria Babini, Alessandra Paggioro, Vieri Parrini, Maria Zaccaria, Stefano Clementi, Carmelo Gigliuto, Francesca Facondini, Simonetta Pastorini, Susanna Munaron, Italo Calamai, Anna Bocchi, Adele Adorni, Maria Grazia Bocci, Andrea Cortegiani, Tariana Casalicchio, Serena Melia, Elia Graziani, Massimo Barattini, Elisabetta Brizio, Maurizio Rossi, Michael Hahn, Hans Flattens, Nicolai Kemmerer, Hans Frank Streiter, Knut Dybwik, Terje Legernaes, Pål Klepstad, Even Braut Olaussen, Knut Inge Olsen, Ole Marius Brresen, Geir Bjorsvik, Finn H. Andersen, Sameer Maini, Lutz Fehrle, Mirosław Czuczwar, Paweł Krawczyk, Mirosław Ziętkiewicz, Łukasz R. Nowak, Katarzyna Kotfis, Katarzyna Cwyl, Ryszard Gajdosz, Jowita Biernawska, Romuald Bohatyrewicz, Ryszard Gawda, Paweł Grudzień, Paweł Nasiłowski, Natalia Popek, Waldemar Cyrankiewicz, Katarzyna Wawrzyniak, Marek Wnuk, Dariusz Maciejewski, Dorota Studzińska, Maciej Żukowski, Szymon Bernas, Mariusz Piechota, Wojciech Szczeklik, Ilona Nowak-Kózka, Jakub Fronczek, Marta Serwa, Waldemar Machała, Jan Stefaniak, Maria Wujtewicz, Małgorzata Szymkowiak, Barbara Adamik, Kamil Polok, Anna Włudarczyk, Jacek Górka, Natalia Kozera, Waldemar Goździk, Nuno Catorze, Miguel Castelo Branco, Inês Barros, Nelson Barros, Andriy Krystopchuk, Teresa Honrado, Cristina Sousa, Francisco Munoz, Marta Rebelo, Rui Gomes, Jorge Nunes, Celeste Dias, Ana Margarida Fernandes, Cristina Petrisor, Bodolea Constantin, Vladislav Belskiy, Boris Boskholov, Enver Rodriguez, Sergio Rebollo, Gerardo Aguilar, Gaspar Masdeu, Marián Irazábal Jaimes, Ángela Prado Mira, Maria A. Bodi, Jesus A. Barea Mendoza, Sonia López-Cuenca, Marcela Homez Guzman, Jesús Rico-Feijoo, Mercedes Ibarz, Josep Trenado Alvarez, Rafael Kawati, Joakim Sivik, Jessica Nauska, Daniel Smole, Fredric Parenmark, Johanna Lyrén, Katalin Rockstrohm, Sara Rydén, Martin Spångfors, Morten Strinnholm, Sten Walther, Lina De Geer, Peter Nordlund, Staffan Pålsson, Harald Zetterquist, Annika Nilsson, Karin Thiringer, Mårten Jungner, Björn Bark, Berit Nordling, Hans Sköld, Camilla Brorsson, Stefan Persson, Anna Bergström, Johan Berkius, Johanna Holmström, I. van Dijk, Lenneke E. M. Haas, D. Ramnarain, Tim Jansen, Fleur Nooteboom, Peter H. J. van der Voort, Dylan de Lange, Willem Dieperink, Monique C. de Waard, Annemarie G. E. de Smet, Laura Bormans, Tom Dormans, Ged Dempsey, Shiju J. Mathew, Ashok S. Raj, Irina Grecu, Jason Cupitt, Tom Lawton, Richard Clark, Monica Popescu, Nick Spittle, Maria Faulkner, Amanda Cowton, Esme Elloway, Patricia Williams, Michael Reay, Srikanth Chukkambotla, Ravi Kumar, Nawaf Al-Subaie, Linda Kent, Tiina Tamm, Istvan Kajtor, Karen Burns, Richard Pugh, Marlies Ostermann, Elisa Kam, Helen Bowyer, Neil Smith, Maie Templeton, Jeremy Henning, Kelly Goffin, Ritoo Kapoor, Shondipon Laha, Phil Chilton, Waqas Khaliq, Alison Crayford, Samantha Coetzee, Moira Tait, Wendy Stoker, Marc Gimenez, Alan Pope, Julie Camsooksai, David Pogson, Kate Quigley, Jenny Ritzema, Anil Hormis, Carole Boulanger, M. Balasubramaniam, Luke Vamplew, Karen Burt, Daniel Martin, Irina Grecu, Jayne Craig, John Prowle, Nanci Doyle, Jonathon Shelton, Carmen Scott, Phil Donnison, Sarah Shelton, Christian Frey, Christine Ryan, Dominic Spray, Christine Ryan, Veronica Barnes, Kerry Barnes, Stephanie Ridgway, Rajnish Saha, Linda Kent, Thomas Clark, James Wood, Clare Bolger, Christopher Bassford, Amanda Cowton, John Lewandowski, Xiaobei Zhao, Sally Humphreys, Susan Dowling, Neil Richardson, Andrew Burtenshaw, Carl Stevenson, Danielle Wilcock, Yuiry Nalapko

**Affiliations:** 1 Department of Intensive Care Medicine, Hospital Universitario Sagrat Cor, Viladomat 288, 08029 Barcelona, Spain; 2 Assistance Publique-Hôpital de Paris, Hôpital Saint-Antoine, Service de Réanimation Médicale, 75012 Paris, France; 3 Department of Intensive Care Medicine, Diakonessenhuis Utrecht, Utrecht, The Netherlands; 4 Department of Intensive Care Medicine, Hospital Universitario General de Cataluña, Sant Cugat del Valles, Spain; 5 Department of Anaesthesia and Intensive Care, Haukeland University Hospital, Bergen, Norway; 6 Department of Intensive Care Medicine, University Medical Center, University of Utrecht, Utrecht, The Netherlands; 7 Department of Rehabilitation Hospital Ancelle di Cremona, Cremona, Italy; 8 Geriatric Research Group, Brescia, Italy; 9 Department of Anaesthesia and Intensive Care, Ålesund Hospital, Ålesund, Norway; 10 NTNU, Department of Circulation and Medical Imaging, Trondheim, Norway; 11 Laboratorio di Epidemiologia Clinica, Centro di Coordinamento GiViTI Dipartimento di Salute Pubblica, IRCCS - Instituto di Ricerche Farmacologiche “Mario Negri” Ranica, Bergamo, Italy; 12 Department of Anesthesia and Intensive Care Medicine, Humanitas Clinical and Research Center - IRCCS, Via Alessandro 13 Manzoni, 56, 20089 Rozzano, MI Italy; 13 Department of Biomedical Sciences, Humanitas University, Pieve Emanuele, MI Italy; 14 Department of Anaesthesia and Intensive Care Medicine, Aarhus University Hospital, Aarhus, Denmark; 15 ASST Grande Ospedale Metropolitano Niguarda, Milan, Italy; 16 Department of Cardiology, Pulmonology and Angiology, University Hospital, Düsseldorf, Germany; 17 Mater Misericordiae University Hospital, Dublin, Ireland; 18 Unidade de Cuidados Intensivos Neurocríticos e Trauma, Hospital de São José, Centro Hospitalar de Lisboa Central, Faculdade de Ciência Médicas de Lisboa, Nova Médical School, Lisbon, Portugal; 19 Department of Intensive Care 1K12IC, Ghent University Hospital, Ghent, Belgium; 20 Karolinska University Hospital, Stockholm, Sweden; 21 Department of Anaesthesiology, Pharmacology and Intensive Care, Geneva University Hospitals, Geneva, Switzerland; 22 Intensive Care and Perioperative Medicine Division, Jagiellonian University Medical College, Kraków, Poland; 23 Kardinal Schwarzenberg Hospital, Schwarzach, Austria; 24 St George’s University Hospital, London, UK; 25 Intensive Care Unit General Hospital of Larissa, Larissa, Greece; 26 Sorbonne Université, INSERM, Institut Pierre Louis d’Epidémiologie et de Santé Publique, AP-HP, Paris, France; 27 Department of Intensive Care Medicine, CIBER Enfermedades Respiratorias, Corporacion Sanitaria Universitaria Parc Tauli, Autonomous University of Barcelona, Sabadell, Spain; 28 Department of Clinical Medicine, University of Bergen, Bergen, Norway

**Keywords:** Sepsis, Very old, Intensive care, Severity of illness, Outcome, Survival, Mortality

## Abstract

**Background:**

The number of intensive care patients aged ≥ 80 years (Very old Intensive Care Patients; VIPs) is growing. VIPs have high mortality and morbidity and the benefits of ICU admission are frequently questioned. Sepsis incidence has risen in recent years and identification of outcomes is of considerable public importance. We aimed to determine whether VIPs admitted for sepsis had different outcomes than those admitted for other acute reasons and identify potential prognostic factors for 30-day survival.

**Results:**

This prospective study included VIPs with Sequential Organ Failure Assessment (SOFA) scores ≥ 2 acutely admitted to 307 ICUs in 21 European countries. Of 3869 acutely admitted VIPs, 493 (12.7%) [53.8% male, median age 83 (81–86) years] were admitted for sepsis. Sepsis was defined according to clinical criteria; suspected or demonstrated focus of infection and SOFA score ≥ 2 points. Compared to VIPs admitted for other acute reasons, VIPs admitted for sepsis were younger, had a higher SOFA score (9 vs. 7, *p* < 0.0001), required more vasoactive drugs [82.2% vs. 55.1%, *p* < 0.0001] and renal replacement therapies [17.4% vs. 9.9%; *p* < 0.0001], and had more life-sustaining treatment limitations [37.3% vs. 32.1%; *p* = 0.02]. Frailty was similar in both groups. Unadjusted 30-day survival was not significantly different between the two groups. After adjustment for age, gender, frailty, and SOFA score, sepsis had no impact on 30-day survival [HR 0.99 (95% CI 0.86–1.15), *p* = 0.917]. Inverse-probability weight (IPW)-adjusted survival curves for the first 30 days after ICU admission were similar for acute septic and non-septic patients [HR: 1.00 (95% CI 0.87–1.17), *p* = 0.95]. A matched-pair analysis in which patients with sepsis were matched with two control patients of the same gender with the same age, SOFA score, and level of frailty was also performed. A Cox proportional hazard regression model stratified on the matched pairs showed that 30-day survival was similar in both groups [57.2% (95% CI 52.7–60.7) vs. 57.1% (95% CI 53.7–60.1), *p* = 0.85].

**Conclusions:**

After adjusting for organ dysfunction, sepsis at admission was not independently associated with decreased 30-day survival in this multinational study of 3869 VIPs. Age, frailty, and SOFA score were independently associated with survival.

## Introduction

The proportion of patients aged ≥ 80 years admitted to intensive care units (ICU), so-called Very Old Intensive Care Patients (VIPs), is growing fast due to ageing of the population [1]. Nowadays, VIPs represent 10% to 20% of all ICU admissions [2–7].

Infection is one of the most frequent reasons for acute ICU admission of older patients, with increasing incidences over the last decades [8–13]. Sepsis develops when the host’s response to infection becomes dysregulated and leads to life-threatening organ dysfunction [14]. Older patients account for a small proportion of the overall population, but a large proportion of sepsis cases; about 60% of septic patients are aged > 65 years. The incidence of sepsis increases with age and increases steeply in persons aged ≥ 80 years [8–10]. Very old persons are at particularly high risk due to pre-existing comorbidities, impaired immune function (immunosenescence), sarcopenia, decrease in reserve capacities related to ageing, and many times malnutrition and polypharmacy [8–10, 15]. Moreover, mortality rates in VIPs with sepsis are high, with an estimated ICU mortality of 50% to 60% [6], reaching 92% at 6 months in those with circulatory failure [16]. In addition, survivors are at increased risk of developing cognitive impairment and functional disabilities, estimated at 16% to 40% [17–19].

The relatively high risk of mortality and shorter life expectancy amongst VIPs with sepsis, combined with increasing pressure on healthcare facilities including ICUs, result in uncertainty about the appropriateness of admitting VIPs with sepsis to ICUs, especially if they are frail or have severe comorbidities. Given the goal of long-term survival with a satisfactory quality of life (QoL) according to patients’ expectations, it is often difficult to predict the benefits of ICU treatment in VIPs, [19, 20]. To determine whether VIPs with sepsis should be admitted to ICUs, healthcare providers need more information about outcomes and risk factors [21].

We aimed to determine whether VIPs admitted with sepsis had a different 30-day outcome than VIPs admitted for other acute reasons and to identify potential prognostic factors for 30-day survival.

## Materials and methods

### Study design and setting

The present study is a post-hoc analysis of the VIP1 multinational cohort study [1]. Patients with sepsis were identified as a group of interest and before the end of the VIP1 study, we decided to analyse the cohort of VIPs admitted for sepsis versus VIPs admitted for another acute reason.

In brief, the VIP1 study was a prospective observational study to measure outcomes in patients aged ≥ 80 years in 311 ICUs in 21 European countries. Each participating ICU included the first consecutive 20 VIPs admitted within a 3-month inclusion period; data were collected between October 2016 and May 2017. A website was designed to provide information about the study and to enable data entry using an electronic case record form; the electronic case record form and database ran on a secure server at Aarhus University, Denmark. The study was registered at ClinicalTRials.gov (ID: NCTO3134807).

### Participants

From the original VIP1 study, only acute admissions in patients ≥ 80 years of age were eligible. We excluded patients admitted for postoperative care after planned surgery; all the other 11 reasons for acute ICU admissions were accepted (Additional file 1: Table S1).

### Study variables and data collection

Demographic and clinical characteristics were recorded for all patients, including age, gender, hospital length of stay (LOS) prior to ICU admission, LOS in ICU, SOFA score at admission [22], and frailty measured with the Clinical Frailty Scale (CFS) [23].

The main outcome variable was survival in the 30 days following ICU admission. We also recorded the use of the following: invasive mechanical ventilation, non-invasive ventilation, vasoactive drugs, renal replacement therapies (RRT), and orders to withhold or withdraw life-sustaining treatment (LST).

### Definitions

#### Admission categories

The most appropriate clinical reason for ICU admission was chosen by the site investigator from a predefined list of 11 acute categories (respiratory failure, circulatory failure, combined respiratory/circulatory failure, sepsis, severe trauma without head injury, severe trauma with head injury, isolated head injury, intoxication, non-traumatic brain injury, postoperative care after emergency surgery, or other) (Additional file 1: Table S1).

#### Severe sepsis admission category

Patients were included in sepsis category according to clinical criteria.

Clinical criteria adopted since 2015 are ¨suspected or documented infection and an acute increase of ≥ 2 SOFA points (a proxy for organ dysfunction) ¨. It was updated in 2016 in sepsis-3 criteria [14]: ¨Sepsis is a life-threatening organ dysfunction caused by a dysregulated host response to infection. For clinical operationalisation, organ dysfunction can be represented by an increase in the Sequential [Sepsis-related] Organ Failure Assessment (SOFA) score of 2 points or more, which is associated with a in-hospital mortality greater than 10%”.

#### Frailty

It was assessed according to the Clinical Frailty Scale [23]. This scale is composed of nine classes from very fit to terminally ill (Additional file 2: Figure S1). We determined the frailty level present before hospital admission and not affected by the acute illness. Patients were classified according to the CFS as “fit” (CFS ≤ 3), “vulnerable” (CFS = 4), or “frail” (CFS ≥ 5).

### Statistical analysis

No formal sample-size calculation was performed for this observational study. Nevertheless, with the number of subjects included in our sample, to test whether the hazard ratio of septic vs non-septic patients is equal to 1.5, the power is 99. To test whether survival of septic is equivalent to that of non-septic patients, the power is 99.

We compared baseline characteristics, treatment, and outcomes between septic and non-septic VIPs. We expressed categorical variables as frequencies and percentages, and continuous variables as medians and interquartile ranges. There are no missing values amongst the variables used in the analysis, except for 2 patients with missing date of ICU discharge. To compare groups, we used Chi square tests for categorical variables and the Mann–Whitney *U* test for continuous variables.

To study 30-day survival, all patients were censored at day 30. For patients discharged from the ICU and dead at day 30, the precise date of death is unknown; for those, we assumed that the survival time was the middle of the interval between date of discharge and day 30. This mid-point imputation is a simple method to deal with interval-censored data and has been shown to give similar estimates than more advanced methods [24].

Unadjusted survival curves were estimated using the standard Kaplan–Meier estimator and compared between groups by means of a log-rank test.

To estimate associations between variables and survival 30 days after ICU admission, we used a Cox proportional hazard regression model. To check the proportionality assumption for each covariate, we plotted the scaled Schoenfeld residuals against time along with smooth curves and detected no violation of the assumption. Inverse probability weights (IPW) were used to produce survival curves adjusted for covariates [25]. The weights were estimated using the same covariates included in the Cox model, namely frailty, age, gender, type of admission (septic vs. non-septic), and SOFA score to estimate the weights. Informally, each subject is weighted by the inverse of the probability of having sepsis or not conditionally on the covariates.

We also performed a matched-pair analysis. For each septic patient, we identified a non-septic patient of the same age, gender, level of frailty, and an SOFA score equal to that of the septic patient plus or minus 0.1. To estimate associations between sepsis and survival at 30 days after ICU admission in the matched sample, we used a Cox proportional hazard regression model stratified on the matched pairs. We plotted the Kaplan–Meier survival curves of septic and non-septic patients in the matched sample and used the usual log-rank test to compare the curves [26].

*P* values less than 0.05 were considered statistically significant. All analyses were performed with R software, version 3.2.2 (R foundation for Statistical computing).

## Results

### Participants

**Fig. 1:**
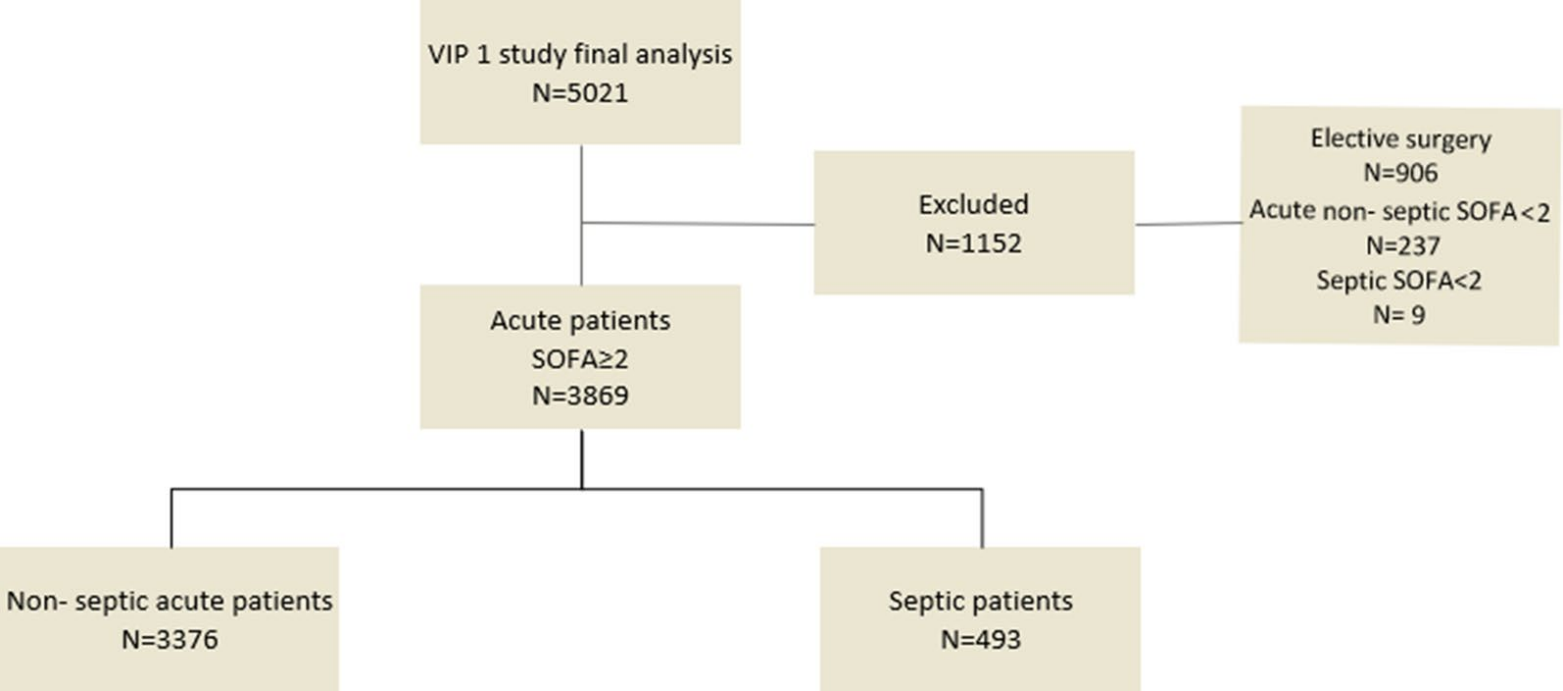
Study flowchart. VIP1 study [1]. Septic patients: patients admitted to ICU for sepsis; non-septic patients: patients admitted to ICU for another acute reason. *SOFA* Sequential Organ Failure Assessment

The VIP1 study included 5132 VIPs; 5021 (98%) completed the 30-day follow-up. Amongst patients who completed the 30-day follow-up, we excluded the 906 (18%) admitted primarily for postoperative care after elective surgery. Moreover, we excluded 246 (4.9%) patients with Sequential (Sepsis-related) Organ Failure Assessment (SOFA) score < 2; thus, we analysed data from 3869 patients (Fig. 1). Regions and countries of the included patients are listed (Additional file 3: Table S2).

### Patient characteristics

We included 3869 VIPs [median age 84 (82–86) years; 2013 (52%) male; median SOFA score 8 (5–11); 47% frail; 32.8% with limitations on LST] admitted as acute patients to 307 ICUs in 21 countries in the context of the multicentre VIP-1 study. LOS before ICU was 1 day (0–3) (see Table 1).

**Table 1:** Comparison of acute patients admitted for sepsis versus acute patients admitted for other reason

Admission category	All acute patients	Other categories	Sepsis	*p* value
*N* (%)	3869 (100%)	3376 (87.3%)	493 (12.7%)	
Age (years)^a^	84 (82–86)	84 (82–87)	83 (81–86)	< 0.0001
Gender (male)	2013 (52%)	1748 (51.8%)	265 (53.8%)	0.4402
Hospital LOS before ICU (days)^a^	1 (0–3)	1 (0–3)	1 (0–3)	0.4600
SOFA score at admission^a^	8 (5–11)	7 (5–11)	9 (6–12)	< 0.0001
ICU LOS (days)^a^	2.96 (1.17–6.81)	2.88 (1.12–6.67)	3.54 (1.5–8)	0.0036
Frailty (CFS)				
Fit (CFS 1–3)	1331 (34.4%)	1166 (34.5%)	165 (33.5%)	0.0737
Vulnerable (CFS 4)	719 (18.6%)	643 (19%)	76 (15.4%)	
Frail (CFS 5–9)	1819 (47%)	1567 (46.4%)	252 (51.1%)	
Fit or vulnerable	2050 (53%)	1809 (53.6%)	241 (48.9%)	0.0568
Frail	1819 (47%)	1567 (46.4%)	252 (51.1%)	
Interventions in ICU				
At least 1 intervention	3196 (82.6%)	2760 (81.8%)	436 (88.4%)	0.0003
No interventions	673 (17.4%)	616 (18.2%)	57 (11.6%)	
Mechanical ventilation	2087 (53.9%)	1853 (54.9%)	234 (47.5%)	0.0024
Non-invasive ventilation	1047 (27.1%)	939 (27.8%)	108 (21.9%)	0.0069
Vasoactive drugs	2265 (58.5%)	1860 (55.1%)	405 (82.2%)	< 0.0001
RRT	421 (10.9%)	335 (9.9%)	86 (17.4%)	< 0.0001
Life-sustaining treatment limitations				
No LST limitations	2601 (67.2%)	2292 (67.9%)	309 (62.7%)	0.0243
LST limitations	1268 (32.8%)	1084 (32.1%)	184 (37.3%)	
Withholding	679 (17.5%)	571 (16.9%)	108 (21.9%)	0.0196
Withdrawing ± withholding	589 (15.2%)	513 (15.2%)	76 (15.4%)	
Outcome				
ICU mortality	1072 (27.7%)	918 (27.2%)	154 (31.2%)	0.0686
30-day mortality	1577 (40.8%)	1357 (40.2%)	220 (44.6%)	0.0687

*LOS* length of stay, *SOFA* Sequential Organ Failure Assessment, *CFS* Clinical Frailty Scale, *RRT* renal replacement therapy, *LST* Life-sustaining treatment

^a^Expressed as median, IQR

No missing values except for length of ICU stay; 2 patients had a missing date of discharge

The median number of patients recruited per country was 143 (range 3–719), and the median number of patients per ICU was 13 (range 1–67).

### Comparison between VIPs admitted for sepsis and those admitted for other acute reasons

Patients admitted for sepsis accounted for 12.7% (493/3869); there was no gender difference, but the sepsis group were younger, had a higher SOFA score on admission, more often received vasoactive drugs and RRT, but were less frequently given mechanical ventilation and NIV. Limitations of life-sustaining treatment (LST) were more frequently performed, and LOS was increased in patients admitted with sepsis (Table 1).

#### Unmatched analysis

No significant differences between groups were observed in survival after ICU admission (*p* = 0.1); survival at day 4 was 78.2% (95% CI 74.6–82.0) in septic patients vs. 82.8% (95% CI 81.5–84.1) in non-septic patients and survival at day 30 was 54.8% (95% CI 50.5–59.5) in septic patients vs. 57.8% (95% CI 56.1–59.5) in non-septic VIPs; HR for septic vs. non-septic patients was 1.13 (95% CI 0.98–1.3), *p* = 0.0986. After adjustment for age, frailty, gender, and SOFA score, sepsis had no effect on survival after ICU admission [HR: 0.99 (95% CI 0.86–1.15), *p* = 0.917] (Table 2A).

**Table 2:** Factors affecting 30-day survival of ICU patients aged ≥ 80 years with SOFA ≥ 2 at admission, multivariate analysis

	HR (95% CI)	*p* value
A. Results of the Cox analysis considering all acutely admitted patients (*n* = 3869)		
Sepsis	0.99 (0.86–1.15)	*p* = 0.9173
Age (per 5-year increase)	1.16 (1.09–1.25)	*p* < 0.0001
Frailty: vulnerable vs. fit	1.16 (1–1.35)	*p* = 0.0556
Frailty: frail vs. fit	1.47 (1.31–1.66)	*p* < 0.0001
Male vs. female	1.16 (1.05–1.28)	*p* = 0.0043
SOFA score (per one-point increase)	1.13 (1.12–1.14)	*p* < 0.0001
B. Results of the Cox analysis considering only acute patients admitted for sepsis (*n* = 493)		
Age (per 5-year increase)	1.33 (1.1–1.61)	*p* = 0.0029
Frailty: vulnerable vs. fit	1.54 (1.02–2.34)	*p* = 0.0416
Frailty: frail vs. fit	1.47 (1.07–2.02)	*p* = 0.0182
Male vs. female	1.12 (0.85–1.47)	*p* = 0.4202
SOFA score (per one-point increase)	1.13 (1.1–1.17)	*p* < 0.0001
C. Results of the Cox analysis considering only acute patients admitted for other reason than sepsis (*n* = 3376)		
Age (per 5-year increase)	1.14 (1.06–1.23)	*p* = 0.0005
Frailty: vulnerable vs. fit	1.11 (0.95–1.31)	*p* = 0.1939
Frailty: frail vs. fit	1.48 (1.31–1.68)	*p* < 0.0001
Male vs. female	1.16 (1.04–1.3)	*p* = 0.0064
SOFA score (per one-point increase)	1.13 (1.12–1.14)	*p* < 0.0001

**Fig. 2:**
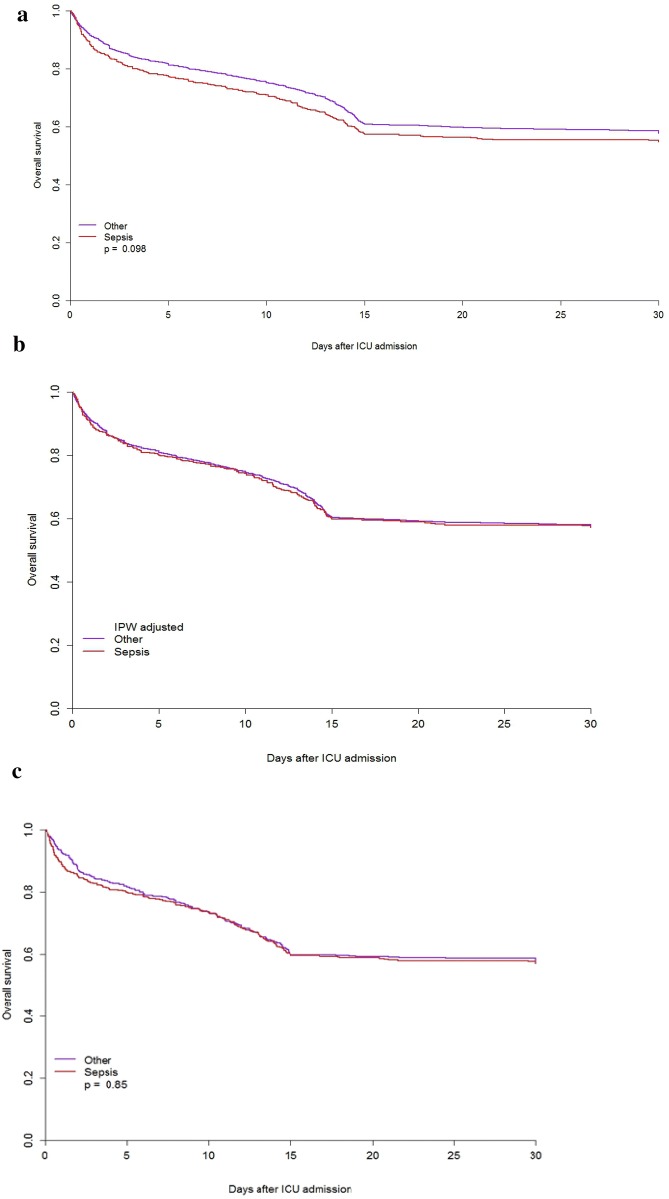
Kaplan–Meyer survival curves in septic and acute non-septic patients. **a** Non-adjusted. **b** Inverse-probability weight (IPW)-adjusted overall survival (the weights were estimated using frailty, age, gender, type of admission, and SOFA score). **c** Matched cohort survival analysis

Inverse-probability weight (IPW)-adjusted survival curves for the first 30 days after ICU admission were similar for septic and non-septic patients [HR: 1.00 (95% CI 0.87–1.17), *p* = 0.947] (Fig. 2b).

Inverse-probability weight (IPW) survival curves for quartiles of the SOFA score in septic and non-septic patients showed no significant differences (Additional file 4: Figure S2).

#### Matched analysis

Likewise, 30-day survival in the matched sample (443 septic patients vs. 824 patients without sepsis, 62 patients had only one match and 55 could not be matched—Table 3) was similar in septic and non-septic VIPs [57.2% (95% CI 52.7–60.7) vs. 57.1% (95% CI 53.7–60.1); HR: 1.02 (95% CI 0.85–1.22), *p* = 0.854] (Fig. 2c).

**Table 3:** Description of the matched sample

Admission category	Other categories	Sepsis	*p* value
*N*	824	443	
Age (years)^a^	82 (81–85)	83 (81–85)	0.5618
Gender (male)	435 (52.8%)	233 (52.6%)	0.9941
Hospital LOS before ICU admission (days)^a^	1 (0–3)	1 (0–3)	0.28
SOFA score at admission^a^	8 (6–11)	8 (6–12)	0.5468
ICU LOS (days)^a^	3.29 (1.33–7.85)	3.88 (1.67–8.53)	0.2454
Frailty (CFS)			0.6963
Fit (CFS 1–3)	273 (33.1%)	144 (32.5%)	
Vulnerable (CFS 4)	105 (12.7%)	64 (14.4%)	
Frail (CFS 5–9)	446 (54.1%)	235 (53%)	
Therapeutic interventions in ICU			
At least one	723 (87.7%)	389 (87.8%)	0.999
Mechanical ventilation	480 (58.3%)	202 (45.6%)	< 0.0001
Non-invasive ventilation	239 (29%)	100 (22.6%)	0.0164
Vasoactive drugs	500 (60,7%)	361 (81.5%)	< 0.0001
RRT	115 (14%)	77 (17.4%)	0.1238
Life-sustaining treatment limitations			
No LST limitations	568 (68.9%)	286 (64.6%)	0.1284
LST limitations	256 (31.1%)	157 (35.4%)	
Withholding	128 (15.5%)	98 (22.1%)	0.0124
Withdrawing ± withholding	128 (15.5%)	59 (13.3%)	
ICU mortality	239 (29%)	126 (28.4%)	0.8841
30-day mortality	337 (40.9%)	187 (42.2%)	0.6942

443 patients with sepsis were matched to 824 patients without sepsis

62 patients had only one match and 55 could not be matched

Survival was similar; sepsis HR 1.02 (95% CI 0.85–1.22), *p* = 0.854

*LOS* length of stay, *SOFA* Sequential Organ Failure Assessment, *CFS* Clinical Frailty Score, *RRT* renal replacement therapy, *LST* life-sustaining treatment

^a^Expressed in median, IQR

### Prognostic factors of survival in all acute admitted patients

In the multivariate analysis, age, frailty, and SOFA score were independently associated with survival, but sepsis was not (Table 2A).

Separate analyses for septic and non-septic patients yielded similar results (Table 2B, C).

A possible centre effect was assessed comparing the log partial likelihood of a model including only sepsis and that of the same model integrating a random centre effect. The *p* value for the random effect was < 0.001 suggesting a significant random effect across centre. We thus built a Cox model using the same variables and integrating a random centre effect. The coefficients and degree of significance of the parameters are comparable to those of the model without random effect (Additional file 5: Table S3).

## Discussion

We found that the 30-day survival rate in patients with sepsis was similar to the survival of patients admitted for another acute reason. Sepsis, after adjusting for organ dysfunction, did not significantly influence. Age, frailty, and SOFA score were the independent factors associated with 30-day survival in patients admitted to ICU for sepsis, similar to all acute VIPs with SOFA ≥ 2. This probably indicates that severity of illness (as expressed by the SOFA score) is the factor that predicts survival independently of whether it is due to sepsis or to other reasons. Therefore, admission for sepsis should not be a factor to limit an ICU admission in this old population.

We collected data from a large cohort in 307 ICUs from 21 European countries. Sepsis was the main reason for admission in 12.7% of the VIPs, a rate similar to those reported in previous studies (9–12%) [8, 15, 19, 20, 27]. Our sample was slightly different to the one analysed in the original VIP1 study, because we excluded the subgroup of patients admitted after planned surgery and compared all acute admissions with the sepsis subgroup. This might explain changes in the results, and in the significance of the lack of variable gender in the present analysis. The original VIP1 paper was designed to study the occurrence of frailty and to assess its impact on 30-day mortality in patients 80 years of age or older admitted to European ICUs. The secondary objective was to report the intensity of care and treatment restrictions whilst in the ICU in this patient group. The original VIP-1 study demonstrated an inverse relation between frailty and 30-day mortality and high mortality rates for VIPs admitted to the ICU with sepsis. We studied and better characterised the subgroup of very old septic patients, identifying the variables associated with outcome, reinforcing that frailty and severity of illness (SOFA) as well as age, and are the determinant factors affecting outcome in VIPs admitted for sepsis. Moreover, we confirmed that sepsis at admission was not a determinant factor on outcome in this population with the analysis of a matched sample with septic and non-septic patients.

Our results are important because relatively few well-designed studies have addressed the impact of sepsis in older patients. Reported ICU-survival rates amongst octogenarians with sepsis vary widely [6, 16, 27, 28], and the risk factors for mortality in these patients have not been completely elucidated. A recent systematic review including 4256 patients aged ≥ 80 years from 18 studies [29] reported mortality rates of 43% in the ICU, 47% in the hospital, and 68% 1 year after ICU admission. Reported rates for 30-day mortality range from 30 [27] to 50% [6, 29].

To our knowledge, this is the first study to compare frequencies of therapeutic interventions, limitations on life-supporting treatments, risk factors for mortality, and outcomes between VIPs admitted with sepsis and those admitted for other acute reasons. In the present study, elective surgical patients were excluded because various other publications [23, 28–32] demonstrated that such patients have a better outcome with much lower mortality rates.

Previous studies reported that limitations on life-sustaining treatment were applied more frequently and earlier in aged patients than in younger patients [27], and moreover, limitations on LST often preceded VIPs’ death in the ICU [27, 33, 34]. However, the intensity of treatment in VIPs has increased over time, and this increase has been associated with a decrease in mortality adjusted for severity [3]. The incidence of LST limitations reported in recent studies ranges from 10 to 27%, being higher in aged patients and reaching 41.6% in very old, frail patients [33–36]. Guidet et al. [37] studied decisions to limit LST in the VIP-1 cohort and demonstrated that acute admission, frailty, age, SOFA score at admission, and country were associated with the application of limitations. We found that patients admitted for sepsis received more therapeutic interventions, mainly vasoactive drugs and RRT. Decisions to limit LST (mainly as withholding therapy) were more common in septic patients (22% vs 17%) and this could be explained because they were frailer and had more organ dysfunction.

Our study’s strengths include its large prospective sample, multicentre design, international participation, and acutely admitted non-septic control group. Furthermore, recruiting all patients prospectively in a period of 8 months reduced time bias.

Our study, however, has several limitations. First, data in VIP1 study were prospectively collected [1] but the data analysis on septic patients was retrospectively done after closure of the database of the original study. Second, admission categories were mutually exclusive and the site investigator in every centre decided to include the patient in one or another category according to the main diagnosis. Severe sepsis was defined according to clinical criteria [14] and we must assume that the individual ICUs appropriately used this definition. However, we cannot fully exclude that some patients may have been misclassified, for example as acute circulatory or respiratory failure. In other words, patients with acute or respiratory failure may also have had a sepsis.

Third, we were not able to analyse the subgroup of patients with septic shock since lactate levels were not available in the registry. Anyhow, 82.2% of the septic patients received vasopressors to maintain a mean arterial pressure of 65 mmHg and mean SOFA at admission was 9. Fourth, the focus of infection was not registered and occurrence of sepsis after ICU admission was neither reported. Fifth, we have no data about patients who were not admitted to the ICU due to triage decisions. Sixth, we did not analyse reasons for LST limitations, because it was not the aim of the study, it is fully analysed in another article [36]. Seventh, the only datum about prior health status recorded was frailty, so no information about comorbidities or previous cognitive status was available. And last, no follow-up of the patients was performed.

Nevertheless, our results provide insight into the outcome and factors associated with 30-day survival in VIPs admitted for sepsis in comparison to VIPs admitted for other acute reasons. The fact that sepsis at admission, after adjusting for organ dysfunction, was not independently associated with survival suggests that the best option today is assessing very old patients according to their age, frailty, and severity of illness, independently of their diagnostic category. Once admitted to ICU, we can establish goals of care and reassess the intensity of therapeutic interventions after a reasonable period of time, according to response to treatment, expected outcomes, and patient/family wishes [38].

## Conclusion

Mortality 30 days after ICU admission is high in very old patients admitted with sepsis. However, we did not find admission for sepsis to be an independent risk factor for decreased survival. Frailty, older age, and higher SOFA score at admission were the significant factors associated with decreased 30-day survival in this population. Therefore, sepsis at admission should not be the only determining factor either in the decision of admission to the ICU or in the establishment of LST in very elderly patients.

Future research is required to optimise care for these patients. We also need more information about long-term survival and quality of life in VIPs admitted for sepsis and a reliable risk prediction model.

## Supplementary information

**Additional file 1: Table S1.** Admission acute categories SOFA ≥ 2.

**Additional file 2: Figure S1.** Clinical Frailty Scale (CFS).

**Additional file 3: Table S2.** Information about region and country of the included patients.

**Additional file 4: Figure S2.** Inverse probability weighted survival curves for quartiles of the SOFA SCORE.

**Additional file 5: Table S3.** Results of the Cox analysis integrating a random centre effect.

## Data Availability

The datasets generated and/or analysed during the current study are not publicly available due privacy issues of the study populations.

## Abbreviations

ICU: Intensive care unit
VIPs: Very Old Intensive Care Patients
RRT: Renal replacement therapy
SOFA: Sequential Organ Failure Assessment
CFS: Clinical Frailty Scale
LOS: Length of stay
LST: Life-sustaining treatment
QoL: Quality of life
