# Supplementary material for: Sepsis at ICU admission does not decrease 30-day survival in very old patients: a post-hoc analysis of the VIP1 multinational cohort study
Source: Ann Intensive Care. 2020 May 13;10:56. doi: 10.1186/s13613-020-00672-w (PMC7221097; doi:10.1186/s13613-020-00672-w)
Supplement: Supplementary file 1 — Additional file 1: Table S1. Admission acute categories SOFA ≥ 2. [file 13613_2020_672_MOESM1_ESM.doc]

**Table S1.** Admission acute categories SOFA≥2

| **Admission categories** | **Number** | **%** |
| --- | --- | --- |
| 1. Respiratory failure | 944 | 24.4 |
| 2. Circulatory failure | 526 | 13.6 |
| 3. Combined 1 & 2 | 481 | 12.4 |
| 4. Sepsis | 493 | 12.7 |
| 5. Severe trauma without head injury | 58 | 1.5 |
| 6. Severe trauma with head injury | 64 | 1.7 |
| 7. Isolated head injury | 113 | 2.9 |
| 8. Intoxication | 13 | 0.3 |
| 9. Non-traumatic brain injury | 290 | 7.5 |
| 10. Postoperative care after  emergency surgery | 373 | 9.6 |
| 11. Other | 514 | 13.3 |

Main reason for ICU admission. One to be chosen as best suited
