# Supplementary figures and images for: Sepsis at ICU admission does not decrease 30-day survival in very old patients: a post-hoc analysis of the VIP1 multinational cohort study
Source: Ann Intensive Care. 2020 May 13;10:56. doi: 10.1186/s13613-020-00672-w (PMC7221097; doi:10.1186/s13613-020-00672-w)

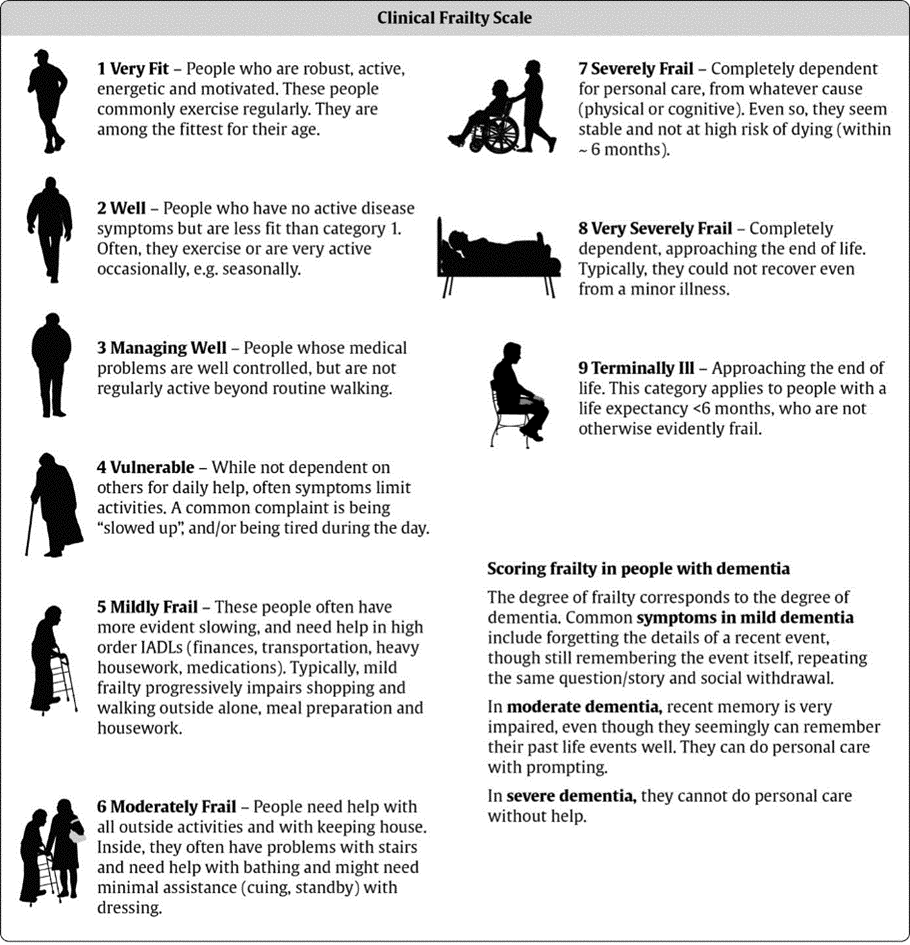


**Figure S1.** Clinical Frailty Scale (CFS); (1, 23)

Supplement: Supplementary file 2 — Additional file 2: Figure S1. Clinical Frailty Scale (CFS). [file 13613_2020_672_MOESM2_ESM.docx]

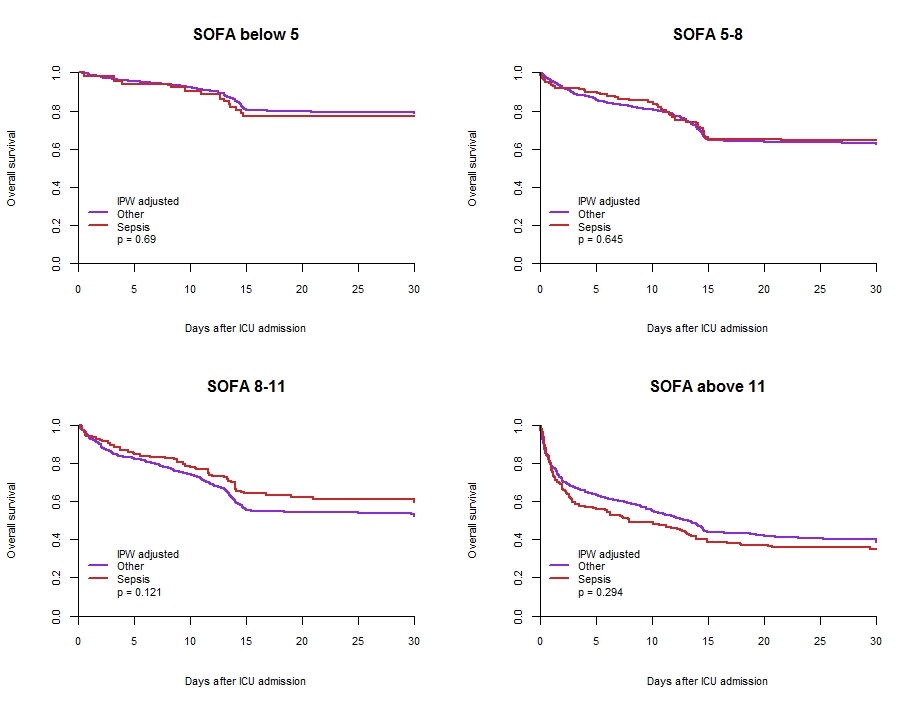

Supplement: Supplementary file 4 — Additional file 4: Figure S2. Inverse probability weighted survival curves for quartiles of the SOFA SCORE. [file 13613_2020_672_MOESM4_ESM.jpeg]
