# Supplementary material for: Sepsis at ICU admission does not decrease 30-day survival in very old patients: a post-hoc analysis of the VIP1 multinational cohort study
Source: Ann Intensive Care. 2020 May 13;10:56. doi: 10.1186/s13613-020-00672-w (PMC7221097; doi:10.1186/s13613-020-00672-w)
Supplement: Supplementary file 3 — Additional file 3: Table S2. Information about region and country of the included patients. [file 13613_2020_672_MOESM3_ESM.doc]

**Table S2.** Information about region and country of the included patients

|  |  | **TOTAL** | **SEPTIC** | **NON-SEPTIC** | **P-VALUE** |
| --- | --- | --- | --- | --- | --- |
|  | **N** | 3869 | 493 | 3376 |  |
| **REGION** |  |  |  |  |  |
|  | East | 459 (11.9%) | 25 (5.1%) | 434 (12.9%) | <0.0001 |
|  | Central | 587 (15.2%) | 72 (14.6%) | 515 (15.3%) |  |
|  | North | 647 (16.7%) | 90 (18.3%) | 557 (16.5%) |  |
|  | South | 1344 (34.7%) | 183 (37.1%) | 1161 (34.4%) |  |
|  | West | 832 (21.5%) | 123 (24.9%) | 709 (21%) |  |
| **COUNTRY** |  |  |  |  |  |
|  | Austria | 52 (1.3%) | 5 (1%) | 47 (1.4%) | <0.0001 |
|  | Belgium | 55 (1.4%) | 8 (1.6%) | 47 (1.4%) |  |
|  | Cyprus | 14 (0.4%) | 0 (0%) | 14 (0.4%) |  |
|  | Czech Republic | 3 (0.1%) | 1 (0.2%) | 2 (0.1%) |  |
|  | Denmark | 92 (2.4%) | 10 (2%) | 82 (2.4%) |  |
|  | France | 347 (9%) | 33 (6.7%) | 314 (9.3%) |  |
|  | Germany | 191 (4.9%) | 25 (5.1%) | 166 (4.9%) |  |
|  | Great Britain | 719 (18.6%) | 102 (20.7%) | 617 (18.3%) |  |
|  | Greece | 143 (3.7%) | 7 (1.4%) | 136 (4%) |  |
|  | Ireland | 113 (2.9%) | 21 (4.3%) | 92 (2.7%) |  |
|  | Italy | 650 (16.8%) | 85 (17.2%) | 565 (16.7%) |  |
|  | Netherlands | 214 (5.5%) | 32 (6.5%) | 182 (5.4%) |  |
|  | Norway | 210 (5.4%) | 31 (6.3%) | 179 (5.3%) |  |
|  | Poland | 228 (5.9%) | 15 (3%) | 213 (6.3%) |  |
|  | Portugal | 194 (5%) | 35 (7.1%) | 159 (4.7%) |  |
|  | Romania | 20 (0.5%) | 0 (0%) | 20 (0.6%) |  |
|  | Russian Federation | 38 (1%) | 2 (0.4%) | 36 (1.1%) |  |
|  | Spain | 153 (4%) | 30 (6.1%) | 123 (3.6%) |  |
|  | Sweden | 345 (8.9%) | 49 (9.9%) | 296 (8.8%) |  |
|  | Switzerland | 75 (1.9%) | 2 (0.4%) | 73 (2.2%) |  |
|  | Ukraine | 13 (0.3%) | 0 (0%) | 13 (0.4%) |  |
