# Supplementary material for: Sepsis at ICU admission does not decrease 30-day survival in very old patients: a post-hoc analysis of the VIP1 multinational cohort study
Source: Ann Intensive Care. 2020 May 13;10:56. doi: 10.1186/s13613-020-00672-w (PMC7221097; doi:10.1186/s13613-020-00672-w)
Supplement: Supplementary file 5 — Additional file 5: Table S3. Results of the Cox analysis integrating a random centre effect. [file 13613_2020_672_MOESM5_ESM.docx]

**Table S3**. **Results of the Cox analysis integrating a random centre effect**

| **Variable** | **HR (95%CI)** | **p-value** |
| --- | --- | --- |
| Sepsis | 0.9 (0.77-1.05) | 0.1800 |
| Age (five years increase) | 1.18 (1.1-1.27) | <0.0001 |
| Vulnerable vs fit | 1.17 (1-1.36) | 0.0560 |
| Frail vs fit | 1.53 (1.35-1.73) | <0.0001 |
| Male vs female | 1.16 (1.04-1.28) | 0.0065 |
| SOFA score (one-point increase) | 1.16 (1.15-1.18) | <0.0001 |
